# Ontogenetic shift in the energy allocation strategy and physiological condition of larval plaice (*Pleuronectes platessa*)

**DOI:** 10.1371/journal.pone.0222261

**Published:** 2019-09-16

**Authors:** Julien Di Pane, Léa Joly, Philippe Koubbi, Carolina Giraldo, Sébastien Monchy, Eric Tavernier, Paul Marchal, Christophe Loots

**Affiliations:** 1 IFREMER, Channel and North Sea Fisheries Research Unit, Boulogne-sur-Mer, France; 2 UFR 918 « Terre, Environnement, Biodiversité », Sorbonne Université, Paris, France; 3 LOG—Laboratoire d’Océanologie et Géosciences, Wimereux, France; Australian Bureau of Agricultural and Resource Economics and Sciences, AUSTRALIA

## Abstract

Condition indices aim to evaluate the physiological status of fish larvae by estimating both the level of starvation and potential of survival. Histological indices reveal direct effects of starvation whereas biochemical indices such as lipid classes or RNA:DNA ratios are used as proxies of condition, giving information on the amount of energy reserves and growth rate, respectively. We combined these three indices to evaluate ontogenetic variations of growth performance, lipid dynamics and nutritional condition of plaice larvae caught in the field during winter 2017 in the eastern English Channel and the Southern Bight of the North Sea. RNA:DNA ratios showed that larvae at the beginning of metamorphosis (stage 4) had a lower growth rate than younger individuals (stages 2 and 3). A significant increase in the proportion of triglycerides also occurred at stage 4, indicating energy storage. Histological indices indicated that most of the larvae were in good condition, even younger ones with low lipid reserves. There was, however, an increase in the proportion of healthy individuals over ontogeny, especially with respect to liver vacuoles which were larger and more numerous for stage 4 larvae. Combined together, these condition indices revealed the ontogenetic shift in the energy allocation strategy of plaice larvae. Young larvae (stages 2 and 3) primarily allocate energy towards somatic growth. The decrease in growth performance for stage 4 was not related to poor condition, but linked to a higher proportion of energy stored as lipids. Since the quantity of lipid reserves is particularly important for plaice larvae to withstand starvation during metamorphosis, this could be considered as a second critical period after the one of exogenous feeding for larval survival and recruitment success.

## Introduction

Marine fish population renewal and its fluctuations between years are mainly influenced by survival rates experienced by early life stages (ELS) [1]. This assumption would mean that the number of individuals reaching adulthood is directly and mainly linked to the number of the young-of-the-year survivors. Recruitment hypotheses focusing on survival of ELS were synthesized by Somarakis et al. [2]. Hjort [3] proposed the “critical period” hypothesis to explain recruitment variability, which is defined as the period when larvae face the highest peak of mortality. The critical period hypothesis is related to the importance of food availability during the transition between endo- and exogenous feeding. More recent hypotheses incorporate mechanisms related to feeding success [4], larval retention [5,6] and growth rate [7]. Thus, physical and trophodynamic processes, and the interaction between the two, make the survival rate of the planktonic pelagic larval phase particularly low, on the order of 0.1% [1,8]. Important mortality during ELS also occurs during other transitional stages such as post-settlement processes [9–11], where juveniles’ survival depends on habitat availability and quality, which are known to be essential in the sustainability of fish populations [12].

Flatfishes experience drastic behavioural and anatomical changes from pelagic and bilaterally symmetrical larvae into asymmetric benthic juveniles [13]. This transitional stage, called metamorphosis, is accompanied by settlement in nursery areas [14]. It is during this transition towards nearshore nursery grounds that the survival of flatfish ELS relies on favourable transports [15–17] and becomes density-dependent [18–21]. Hence, the recruitment variability of flatfish depends on the survival of both larval and early juvenile stages making these development phases bottlenecks of fish populations [8,22]. Prey availability is thought to be one of the main factors limiting the carrying capacity of flatfish nursery grounds (e.g [23–27]), including plaice (*Pleuronectes platessa*) (e.g [28–32]). Thus, post larvae reaching the coasts in a poor condition would be less able to handle competition for food in their new habitat, while individuals with large amounts of energy reserves will be less impacted by starvation. The study of larval condition is therefore an important issue in fisheries biology and ecology.

Methods available to estimate the trophic and physiological condition of fish larvae have been largely reviewed by Ferron and Leggett [33] and Gisbert et al. [34]. Condition indices aim to integrate the physiological status of fish larvae by estimating both the level of starvation and the potential for survival. Direct methods such as histology reveal direct effects of starvation on the digestive tract and associated organs [35–38], whereas biochemical indices (indirect methods) are used as proxies of condition.

Nucleic acids are involved in protein anabolism affecting larval growth [39]. Thus, the RNA:DNA ratio (RD) is related to growth on the principle that while the amount of DNA in a cell is considered constant (often used to define the number of cells in a larva), the amount of RNA is proportional to the level of protein synthesis [39]. Accordingly, organisms in good condition tend to have higher RDs than those in poor condition [40]. The link between growth and condition is also supported by studies that show a decline in RD during starvation (e.g [41–47]). However, variations in RDs can also reflect some life stages and species energetic strategies. For example, individuals can compensate for extended periods of food deprivation either by catabolizing energy reserves in liver tissues [48] or conversely by anabolizing them in prevention, leading to a decrease in the growth rate [49–51].

In the liver, energy is mainly stored as glycogen and lipids [52]. In fish, the use of glycogen as an energy source during fasting appears to be limited and highly variable and most species rely on lipid stores during food deprivation [53]. Liver lipid reserves can be observed through histological sections in the form of vacuoles inside the hepatocytes and their number and size can vary depending on the nutritional status [54]. They represent the first histological criterion responding to food intake and deprivation [55].

Because lipids represent the main form of energy reserves, total lipid content has also been used to define larval condition (e.g [56–58]). Furthermore, storage-lipids are predominantly in the form of triacylglycerol (TAG) [59,60]. During food deprivation, while TAG is catabolized, membrane lipids, such as cholesterol (Chol), remain constant [61]. It is on this principle that Fraser [62] developed the TAG:Chol index to investigate the amount of energy reserves and to be able to compare individuals of different biomass. However, like RD, low values of TAG:Chol do not necessarily mean a poor condition. Larvae with low TAG:Chol values have low energy reserves and lower potential to withstand starvation events [63]. However, in favourable environments with good prey densities, such individuals could still have good probabilities of survival.

In this study, we combined biochemical (RD and TAG:Chol) and histological condition indices to evaluate the ontogenetic variations in growth performance, lipid dynamics and nutritional condition of plaice larvae. The aim was to evaluate growth rates of different development stages of plaice larvae caught *in situ*. By using neutral lipids, we investigated the proportion of energy reserves available. Subsequently, histological observations were made in order to integrate the larval condition. Finally, we compared the results obtained by these different indices to unravel the energy allocation strategy of plaice larvae during their ontogeny.

## Materials and methods

### Sampling strategy

**Fig 1:**
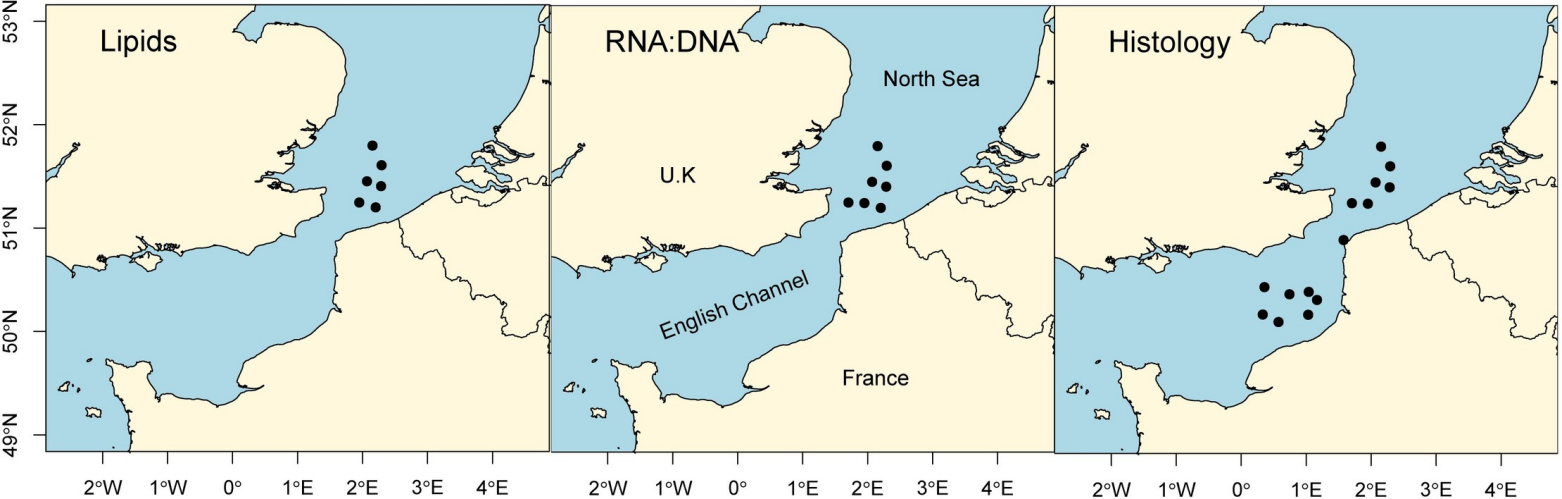
Sampling stations where larvae were collected for analysis.

Plaice larvae were sampled during the International Bottom Trawl Survey in 2017 which took place between the 20^th^ January and the 10^th^ February on board the R/V “Thalassa”. This survey covered the eastern part of the English Channel as well as the Southern Bight of the North Sea (Fig 1).

Fish larvae were caught at night using a midwater ring net (2 m diameter, 13 m long, 1.6 mm mesh size except the last meter which was 500 μm). The net was deployed through a double-oblique tow between the surface and five meters above the seabed during 10 minutes at a speed of 3 knots. The content of the net was sorted immediately on board to collect plaice larvae. Larvae were either preserved in cryotube vials in liquid nitrogen for biochemical analyses or in Bouin’s solution for histological purposes. After 48 hours of fixation in Bouin, larvae were rinsed with ethanol 70% and then preserved in vials with ethanol. Cryotube vials were preserved at—80°C at the laboratory.

Species identification was checked under a stereomicroscope back at the laboratory. Larvae were staged following Shelbourne [64] and Ryland [65]: stage 1- yolk-sac larvae; stage 2- yolk sac exhausted and notochord straight; stage 3- caudal extremity of notochord bent and eyes symmetrical; stage 4: the eyes start to be asymmetrical indicating the beginning of metamorphosis; stage 5- the left eye on or beyond the edge of the head.

The standard length (SL) of each larva was measured to the nearest millimetre. Numbers of individuals used for each analysis are presented in Table 1.

**Table 1:** Number of individuals by development stage used for each analysis.

Stage	2	3	4
Standard length	150	462	67
RD	33	18	29
Total lipids content	17	43	7
TAG:Chol	10	30	8
Histological indices	39	32	23

### Analyses

#### RNA:DNA ratio

Each frozen larva was crushed by a vortex mixer in vials filled with 200 μL Tris-SDS (4°C) and containing some glass microbeads. Extraction of DNA and RNA was done under cold condition using ice-blocks following Yandi and Altinok [66]. Nucleic acids concentration was measured with a Qubit 2 fluorometer (Thermo Fisher, Waltham, MA, USA) using RNA DNA HS assay Kits (Invitrogen, Life Technologies) following Denis et al. [46]. RD values were obtained and the instantaneous growth rate (G_i,_ days^-1^) was calculated for each larva following Buckley et al. [67] and Denis et al. [46] in order to correct for the effect of temperature.

$$G_{i}=0.0145\times RD+0.0044\times \left(RD\times T\right)-0.078$$

Where RD corresponds to the RNA:DNA ratio calculated and T to sea surface temperature (°C) of the sampling site where the larvae were caught. A G_i_ value of 0 means that there is no growth and a larva with a value of 1 doubles its biomass per day.

#### Lipids analyses

Larvae dedicated to lipid analyses were first freeze-dried (-20°C) and their dry weight (DW) was measured with an ultra-precision scale (10^−5^ g). Lipids were then extracted from freeze-dried larvae following a modified Folch method with 2:1 chloroform–methanol containing 0.01% butylated hydroxytoluene (v/v/w) and 0.88% NaCl, for final proportion of chloroform/ methanol/water of 8:4:3 [68]. The lipid phase was filtered, collected, and dried with anhydrous sodium sulfate and evaporated under nitrogen to obtain estimates of the total lipid weight. Total lipids (TL) were stored at -80°C. Lipids classes were quantified using TLC–FID (thin layer chromatography–flame ionization detector in an Iatroscan MK-6s instrument—Analyser Iatron Laboratories, Tokyo, Japan). TL (30 μg/ml diluted on hexane) were loaded on to the chromarods (Type S5) using an automatic spotter (NTS 3000) set up to deliver 2 μl. Each sample was analysed in triplicate. Lipid classes were separated depending on their polarity by solvent baths. For neutral lipids migration (Chol and TAG), chromarods were sequentially developed using n-hexane:benzene:formic acid 80:20:1 (v/v/v) during 30 minutes followed by n-hexane:diethylether:formic acid 97:3:1.5 (v/v/v) for 29 minutes. After developing, chromarods were dried for 5 minutes in an oven at 110°C and then immediately scanned with the TLC-FID to detect and quantify the different lipid classes. ChromStar CHS-1 Software was used to calculate pick areas and retention times. Amount of TL was post calculated.

#### Histology

Standard histological techniques were adapted from Martoja and Martoja [69]. Plaice larvae were dehydrated by alcohol baths of 15 minutes that were progressively more concentrated (70%, 95%, 100%). Each bath was replicated three times. Larvae were then cleared in three xylol baths of 15 minutes each. They were then immersed in two successive paraffin baths at 60°C during two hours each, before being embedded in paraffin blocks. Sagittal sections of 7 μm thick slices were mounted, dewaxed and rehydrated by successive bath of 5 minutes replicated three times of xylol, ethanol 100%, 95%, 70% and distilled water. Finally, slides were stained with Groat’s hematoxylin (2 minutes) and picro indigo carmine (15 seconds), then flushed three time in absolute ethanol baths, one time in xylol bath, and mounted with cover slips.

Slices were observed under optical microscope and a grade of condition was attributed to each larva. Grades were defined based on an extensive review of the literature describing for fish larvae the patterns of histological degradations of criteria commonly used for intestine (midgut and hindgut), pancreas and liver [35,37,70–76]. three grades of condition were then defined as follows:

**Grade 3** (healthy): Epithelial cells of hindgut and midgut are large and convoluted with good integrity and many microvilli. Cytoplasm of hepatocytes contains many textures and nucleus is lateral and reduced with distinct nucleoli. Liver’s vacuoles are numerous, wide and liver cells are attached. Tissue organisation of the pancreas is joined with well-defined acini (symmetrical circular shape) and distinct nuclei in basal position.

**Grade 2** (intermediate): Beginning of starvation. Wide liver vacuoles have disappeared, leading to a central position and a larger size of hepatocytes nucleus. Epithelial cells of hindgut and midgut are reduced with moderate microvilli and some detachments are observable. Hepatocytes’ nucleus is dark and grainy while cytoplasm appears homogeneously granular. Pancreas can show some detachments with acini weakly distinct as well as their nucleus.

**Grade 1** (emaciated): This grade is mainly represented by individuals with poor integrity of the pancreas having large separations as well as the epithelial cells of guts and their brush border. Epithelial cells, especially for the hindgut, are also very reduced or even cuboid. Others criteria associated to a lesser extent are acini’s nucleus which are indistinct and potentially pycnotic. Liver’s cells are disjunctive leading to a loss of lamellar structure, with darkly stained nucleus and hyaline cytoplasm.

Additionally, a score was also given to the liver vacuoles state. Score 1 indicated absence of vacuoles; score 2 corresponded to a liver with rare and scattered vacuoles, while score 3 individuals showed numerous and wide vacuoles.

#### Data analyses

Statistical analyses were performed under the R software (R core Team, 2019) with a threshold of significance fixed at 5%. Size ranges for each development stage were calculated on frozen larvae. Before comparison, normality (Shapiro’s test) and homoscedasticity (Fisher’s test) of raw data were checked. When parametric test application conditions were met, an ANOVA followed by a post hoc HSD Tukey test were performed. For non-Gaussian data, a log transformation was performed. G_i_, TL and TAG:Chol values were compared between development stages. Additionally, linear models were performed on indices depending on size data. Normality of residuals was checked to evaluate model relevance. Analysis of covariance (ANCOVA) was conducted in order to tease out the effect of stage and body length on TL and TAG:Chol values. For histological indices, a graphic description of the proportion of each grade and liver vacuoles score was made by development stage. Since the sampling area of larvae used for histological samples was more extended than for others indices (it also includes samples from the eastern English Channel), a Pearson’s Chi^2^ test was applied to test dependency of histological grades and development stages between the eastern English Channel and the Southern Bight of the North Sea. Finally, in order to combine all indices, three quantiles (low, medium and high) were defined for TAG:Chol and G_i_ values. The number of individuals by stage in each quantile and histological grades and vacuoles scores were assessed. A correspondence analysis (CA) was then performed and associations between rows (stages) and columns (indices levels) were observed.

## Results

### Size distribution per development stages

**Fig 2:**
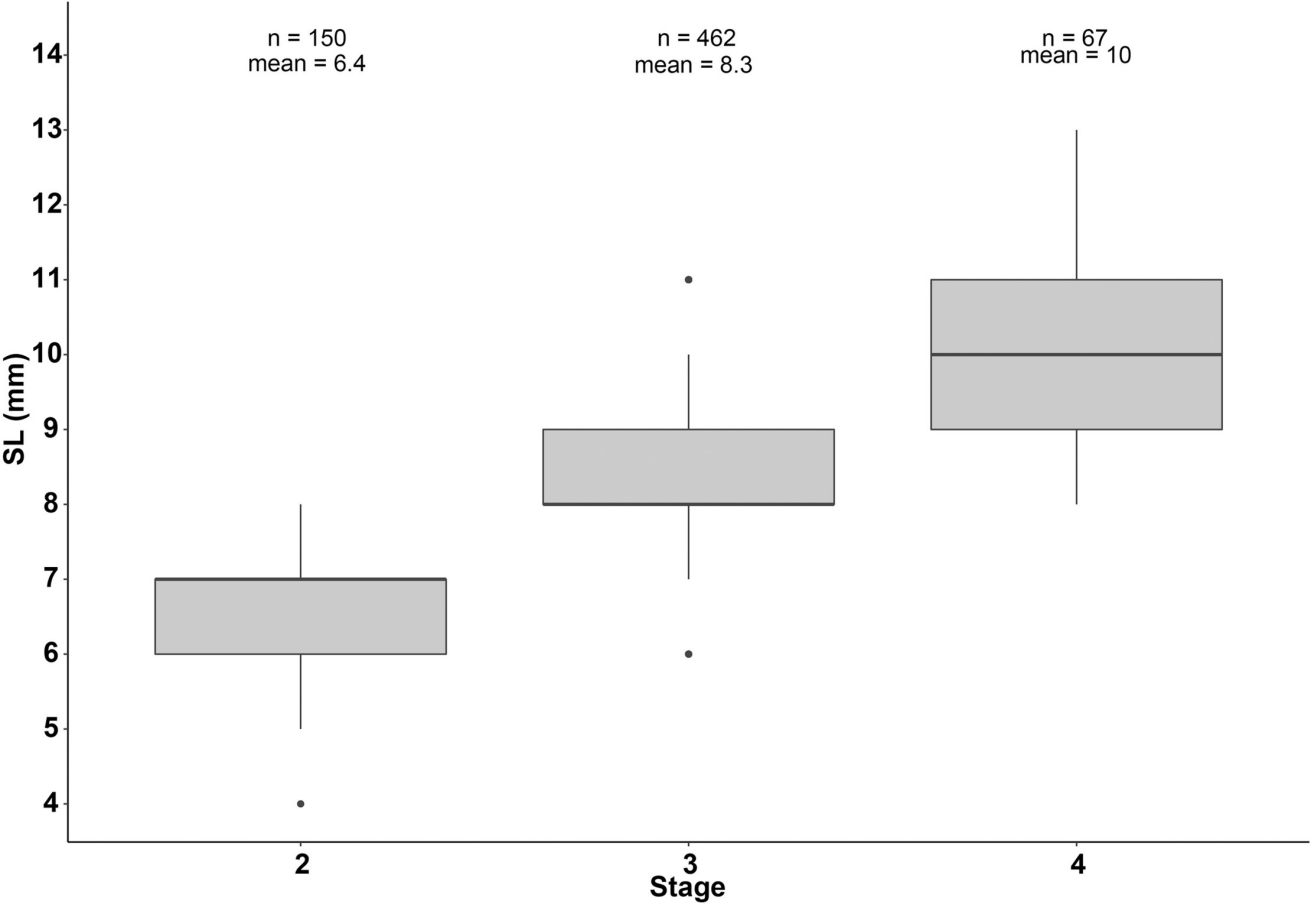
Range of SL observed for each development stage.

A total of 679 plaice larvae were measured with size ranging from 4 to 13 mm (mean = 8.1 mm ± 1.4 mm) corresponding to individuals of stages 2, 3 and 4. No stage 1 or 5 was caught during the survey. Transition between stage 2 and 3 was observed at a SL between 7 and 8 mm. Larvae of stage 4 were observed from 9 mm (Fig 2).

### Biochemical indices

**Fig 3:**
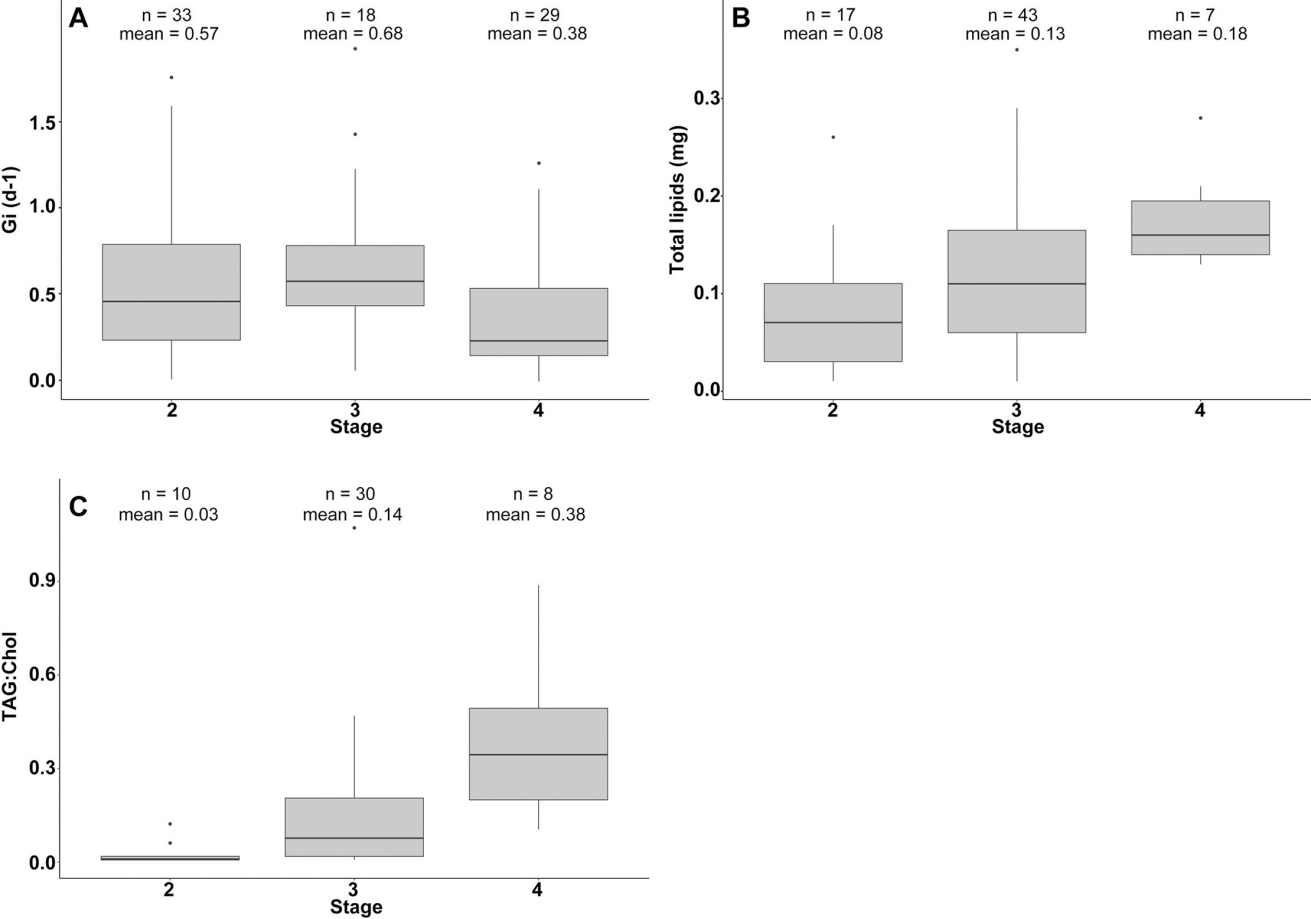
Biochemical indices according to development stage of plaice larvae. A: instantaneous growth rate (G_i_, days^-1^); B: Total lipids amount (TL; mg); C: TAG:Chol index.

The G_i_, total lipids content and TAG:Chol index were calculated for each development stage (Fig 3).

The G_i_ varied from 0 to 1.92 with a mean of 0.52 ± 0.43 days^-1^ (*n* = 80). Medians or means per stage were always positive and non-null (Fig 3A). G_i_ was significantly different between stages (ANOVA, *F(2*,*77)* = 3.5; *P* = 0.03). Post hoc Tukey tests found a higher G_i_ for stages 3 compared to stage 4 (*P* = 0.04). G_i_ of the stage 2 showed no significant differences with the stage 3 (*P* = 0.58) and stage 4 (*P* = 0.16).

TL varied from 5.3 to 57% of dry weight. TL increased with size following the linear regression: TL = 0 + 0.023*SL. However, despite a significant slope, the correlation was weak (LM, *P* < 0.01; *R*^*2*^*adj* = 0.1). TL varied between larval stages (ANOVA: *F*(2,64) = 5.3; *P* < 0.01) (Fig 3B). A post hoc Tukey test showed that there were no significant differences of TL between stage 2 and 3 (*P* = 0.06) and between stage 3 and 4 (*P* = 0.2). Stage 4 had a significantly larger amount of TL than stage 2 (*P* < 0.01). Mean TL varied among developmental stages even when SL was taken into account (ANCOVA: *F*(2, 62) = 8.07; *P* < 0.01). The removal of SL and interaction between stage and SL did not reduced the explanatory power of the model (*P* = 0.1).

Proportions of the main lipid classes were calculated. Plaice larvae were mainly composed by structural lipids with polar lipids (mainly phosphatidylcholine and phosphatidylethanolamine) in relatively stable proportions among individuals and representing 91% (± 3.4%) of total lipids, followed by Chol (6.9 ± 1.6%). TAG (energy reserves) represented only 1.1% and were highly variable between individuals (sd = 1.6%). TAG:Chol ratio ranged from 0 to 1.1 and increased significantly with size following the formula: log(TAG:Chol) = -4.02 + 0.33 * SL (*P* < 0.01; *R^2^adj* = 0.42). The ratio was also represented and tested between stages (Fig 3C). An ANOVA followed by a post hoc Tukey test showed significant differences when comparing stages (*F*(2,45) = 14.33; *P* < 0.01) with differences of TAG proportion between all stages (P< 0.05). Mean TAG:Chol varied among developmental stages even when SL was taken into account (ANCOVA. *F*(2, 42) = 7.8; *P* < 0.01). The comparison with the simplified model with just stage indicated that removing SL and their interaction did not cause a significant reduction of the explanatory power (*P* = 0.16).

### Histology

#### Histological condition index

**Fig 4:**
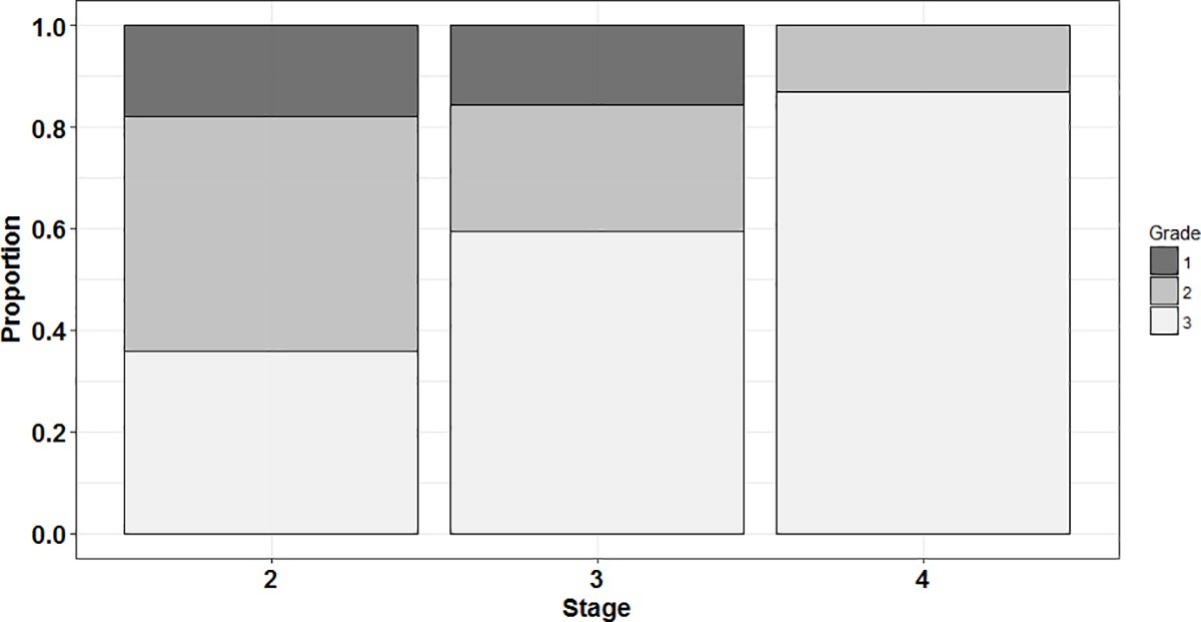
Distribution of histological grades for each stage. Grade 1 (emaciated), 2 (intermediate) and 3 (healthy).

A grade of condition was attributed to each larva, classifying the level of tissue degeneration related to the level of starvation. There was no geographical effect on the histological grades (Pearson’s Chi^2^ test: *df* = 2; *P* = 0.97) nor on the proportions of different developmental stages (*P* = 0.91) between the eastern English Channel and the Southern Bight of the North Sea. Proportions of grades for the different stages were calculated and are depicted in Fig 4.

Overall, grade 1 (emaciated) represented 12.8% of the larvae analysed, grade 2 (intermediate) 30.8% and grade 3 (healthy) 56.4%. Stage 2 larvae comprised 17.9% of grade 1, 46.1% of grade 2 and 36% of grade 3 individuals. For stage 3, grade 1 was found in 15.6% of larvae analysed against 25% of grade 2 and 59.4% of grade 3. Concerning stage 4, no grade 1 was found and 13% of them were grade 2. Grade 3 represented 87% of stage 4 individuals analysed.

#### Hepatocytes vacuoles

**Fig 5:**
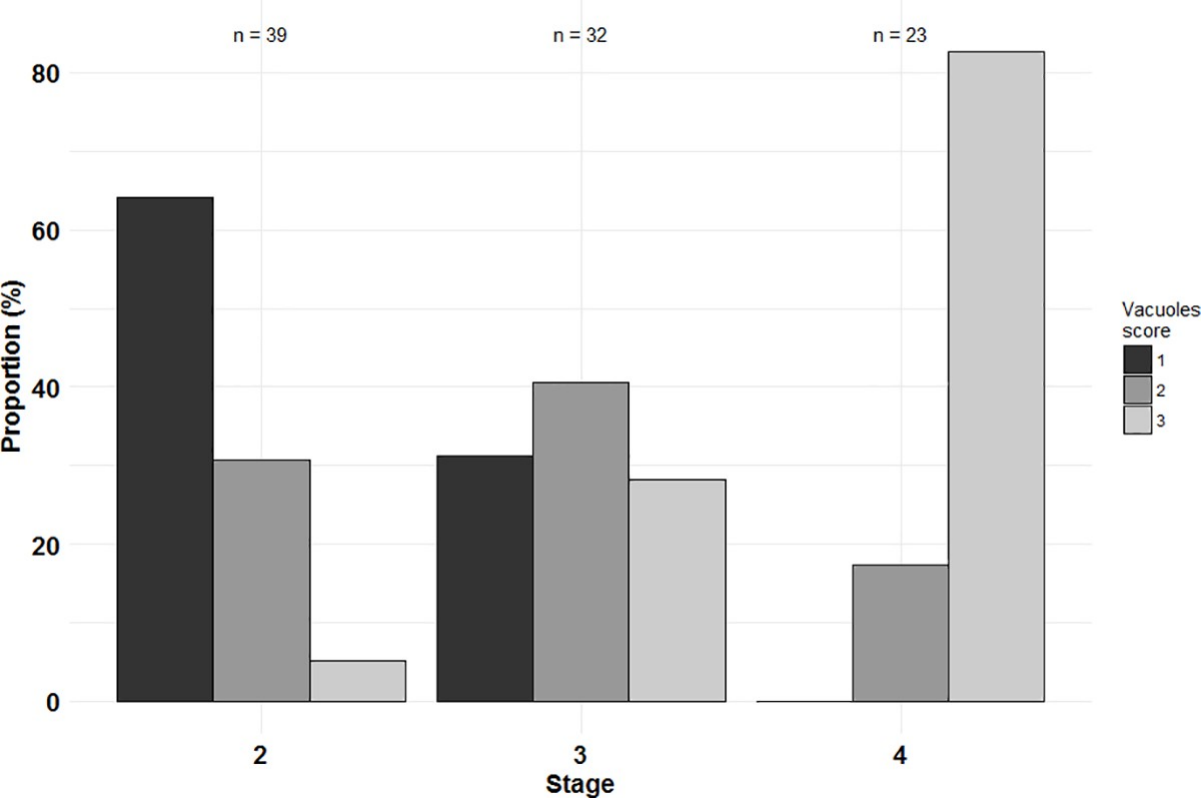
Proportion of liver vacuoles scores according to stages. **1**: Absence of vacuoles; **2**: rare and dispersed vacuoles; **3**: numerous and wide vacuoles.

Size and number of vacuoles in the liver were scored. Results by stage are represented in Fig 5.

**Fig 6:**
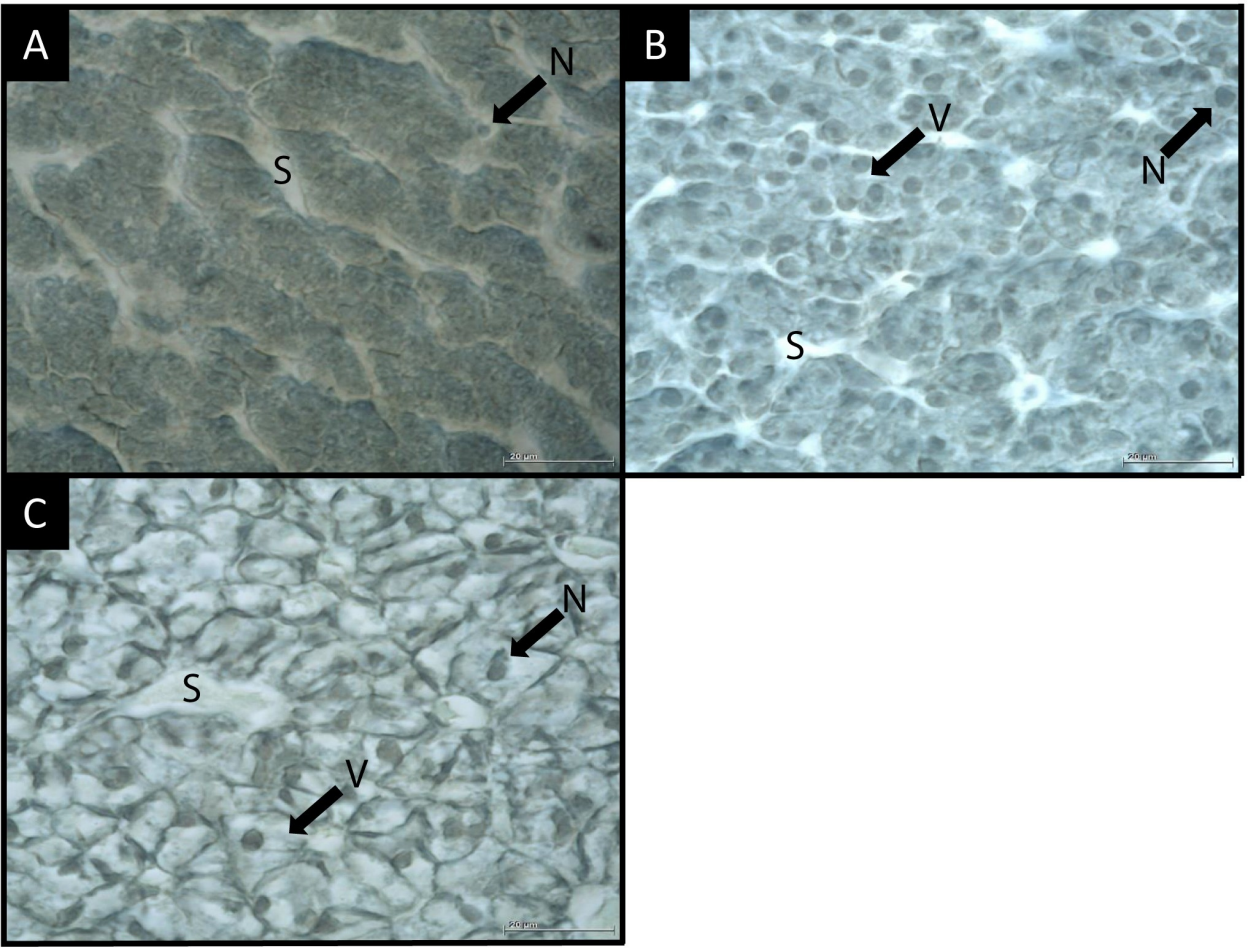
Sagittal histological section of larvae liver plaice. Magnification X1000; oil immersion. **A**: Score 1. Absence of vacuoles. Hepatocytes are small with small and mostly indistinct nuclei. **B**: Score 2. Vacuoles are rare and scattered. Hepatocytes are medium and distinct. Nucleus is central with extended nucleoli. **C**: Score 3. Vacuoles are numerous and wide. Hepatocytes are large and distinct. Nucleus is lateral with reduced and distinct nucleoli. S: Sinusoids; V: Vacuoles; N: Nucleus.

Stage 2 larvae had a proportion reaching 64.1% of individuals with liver vacuoles of score 1 (absence of vacuoles; Fig 6A), 30.8% of score 2 (rare and dispersed vacuoles; Fig 6B) and 5.1% of score 3 (numerous and wide vacuoles; Fig 6C). Stage 3 individuals had 31.2% of score 1, 40.6% of score 2 and 28.2% of score 3. All stage 4 larvae presented vacuoles in the liver (scores 2–3), with 82.6% of individuals with numerous and wide vacuoles (score 3).

### Comparison of indices

The different indices were coupled to highlight physiological characteristics of each development stage. Three classes based on quantiles were defined for Gi and TAG:Chol values to compare those indices based on equifrequent classes (Table 2).

**Table 2:** Limits of classes based on quantiles for TAG:Chol and growth rate indices.

	Low	Medium	High
TAG:Chol	[0_0.02)	[0.02_0.15)	[0.15_1.07]
Gi (Days^-1^)	[0_0.24)	[0.24_0.63)	[0.63_2.85]

**Fig 7:**
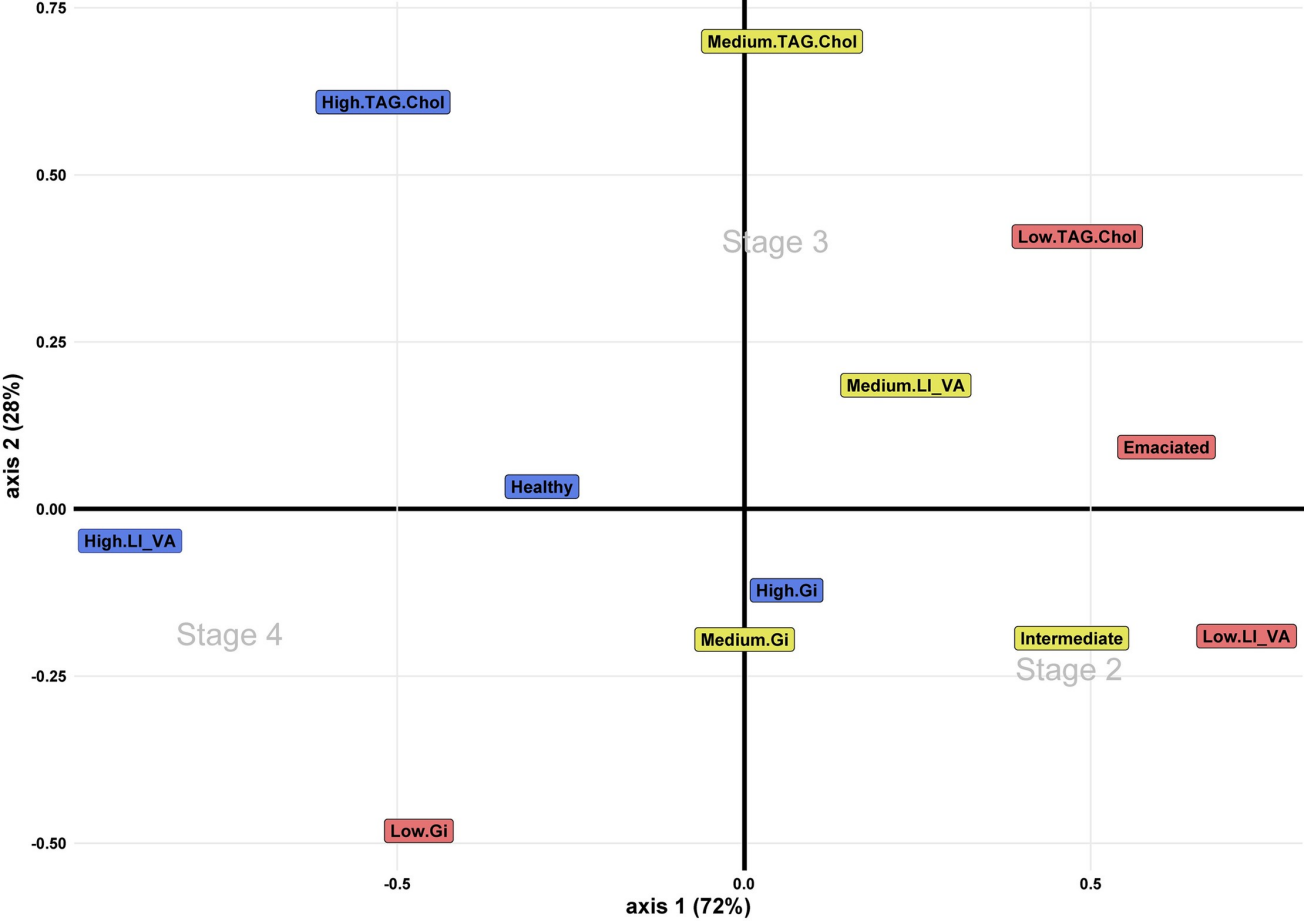
CA of the proportion of individuals of each stage on the three quantiles of Gi and Tag:Chol indices and on the three histological grades of condition and liver vacuoles scores. Colour code depicts quantiles and histological indices levels.

Number of individuals by stage in each Gi quantiles, TAG:Chol classes, histological grades and liver vacuoles scores were computed and a CA was performed (Fig 7).

Both axes explained 100% of the variance. Stages 2 and 4 had a better quality of representation on axis 1 (cos^2^: 0.79 and 0.94 respectively) whereas stage 3 was associated to axis 2 (cos^2^ = 0.99). There was a trend in the repartition of stages along the first axis with stage 2 on the right, stage 3 in the middle and stage 4 on the left of the plane. On the first axis, the majority of medium and low quantiles and histological scores were on the positive part close to stage 2. Only “Low.Gi” was on the negative area, close to stage 4. Conversely, all high quantiles and histological scores were on the negative area of the first axis.

## Discussion

As with other flatfish, plaice experiences drastic changes during its larval development in terms of morphology [13], behaviour [77] and living environment [21], either at the start of the exogenous feeding (stage 2) or when larvae enter stage 4 (with fully developed fins, increased body height and the beginning of eye migration). In this study, histological and biochemical indices were used and compared in order to determine the physiological status and development strategy of wild caught plaice larvae during ontogeny.

Size ranges by development stage were obtained from thawed larvae. From our observations, stage 4 larvae were observed from a standard length of 9 mm. This size corresponds to the start of the metamorphosis and corroborates Ryland [65]. However, the age and size of the beginning of metamorphosis can vary among individuals because of environmental influences including response to nutritional condition [78] and temperature [79–81]. Using otolith microstructure, Comerford et al. [82] showed that hatching dates, larval duration and time of settlement were different among the three regions they compared (southern North Sea, Irish Sea and west of Ireland) because of their differences in temperature. Thus, temperature influences growth rate, usually positively, as it has been shown for plaice larvae [82], and ultimately size at metamorphosis. In this study, size distributions allowed for an accurate distinction of the different stages of development with low overlap. This homogeneity of size at stage could come from the choice of sampling stations wich limited spatial influence of environmental parameters such as temperature.

Ratio of nucleic acids was investigated to highlight differences of growth rate according to development stages. G_i_ of stage 2 larvae appeared highly variable between individuals and displayed a wide range of values. This resulted in an absence of statistical difference with other stages. Growth rate is positively correlated to nutritional status [43]. Hence, the high variability of G_i_ for stage 2 larvae could be due to the mix of fast-growing healthy individuals and slow-growing starved larvae since stage 2 larvae experience their first exogenous feeding and are highly sensitive to starvation (Hjort [3]). Stage 3 individuals showed the highest mean Gi, whereas larvae of stage 4 displayed the lowest growth rate and were significantly different from the one of stage 3 larvae.

This pattern of growth along ontogeny is in accordance with Christensen and Korsgaard [83], which highlighted a constant growth rate during pre-metamorphosis stages (stage 1 to 3 included) mainly due to hyperplasia (cell proliferation). At the beginning of metamorphosis (stage 4), they found a decrease of growth due to a switch toward hypertrophy (cell enlargement). Such changes in growth continue throughout the transition to the post-metamorphosis stage (stage 5), and have been found to extend to newly settled fish [84]. Even if stage 4 larvae had the lowest growth rate, mean G_i_ did not reach zero, suggesting that there was no cessation of energy allocation towards growth. Hovenkamp [85] showed that during metamorphosis fast-growing individuals of North Sea larval plaice demonstrated higher survival rate. The growth-dependent mortality [86] is a well-known phenomenon, which has been highlighted both in field and laboratory studies. According to this hypothesis, even reduced, growth of plaice larvae in metamorphose could be maintained to reduce risks of predation with a shorter duration of the vulnerable larval period [87–89].

Overall, an increase in TL content and TAG proportion was shown over ontogeny. The increase in TL was expected due to the augmentation of the dominant membrane lipid class (98% of the TL content) related to somatic growth. Reserves lipids such as TAG, that are directly influenced by nutrition also increased during larval development, indicating an increase of energy storage for older larvae. Larvae of stage 2 were characterized by extremely low proportions of TAG, with numerous individuals with no reserves, or in quantities below the detection range of Iatroscan. Under optimal feeding condition in an *ex-situ* experiment, plaice larvae start to accumulate neutral lipids by late stage 2 [50]. Our results suggest that for wild individuals, that might encounter less favourable feeding conditions, reserve lipid deposition starts effectively by stage 3. The highest proportion of TAG was found for stage 4 larvae, indicating overall no poor condition for this stage since energy storage occurs in good health [62]. Brewster [90] hypothesized that flatfishes store energy in the liver in foresight of metamorphosis which could begin only after a sufficient amount of reserves has been created. Even if our study does not allow us to confirm this, the absence of stage 4 larvae without reserves is in line with Brewster hypothesis. Accumulating reserves during pre-metamorphosis stages is in all case vital for plaice larvae since they cease to feed during metamorphosis [91,92].

Histological condition and the amount of liver vacuoles increased during development from stage 2 to stage 4. The liver plays a role as an energy reservoir in fish larvae with important functions in lipids and glycogen storage [93,94]. Storage increase vacuolation of cytoplasm hepatocytes leading to the move of the nucleus to the periphery of the cell [54]. Liver reserves are sensitive to nutritional status and are the first energy sources mobilised at the onset of starvation [55]. For these reasons, scoring level of vacuolation is one of the most accurate histological criteria to detect the beginning of starvation and evaluate physiological and nutritional status of fish larvae. Stage 2 larvae had the higher proportion of individuals in degraded and intermediate condition. Most of them had no vacuoles in the liver. Stage 3 larvae had a similar proportion of larvae in poor condition, but had a higher proportion of healthy individuals with a higher proportion of individuals having large liver vacuoles. No stage 4 larvae where found in poor condition. Moreover, most of these larvae had numerous and wide liver vacuoles. Absence of stage 4 individuals without liver vacuoles corroborates the hypothesis advanced by Brewster [90] on the necessity of stage 3 individuals building up energy reserves before entering metamorphosis. Stage 4 larvae were in better condition than stage 3 ones, but in much lower abundances. Thus, the peak of mortality due to starvation experienced by first feeding larvae (beginning of stage 2) could extend until beginning of metamorphosis.

Taken together, lipid classes proportion, histological observations and growth index allowed the characterisation of the physiological condition of larval plaice throughout ontogeny. While histological indices revealed condition, linked to direct effects of starvation, biochemical indices such as lipid classes or nucleic acids ratios provided information on the amount of energy reserves and growth rate, respectively. When fish begin larval exogenous feeding and until settlement, they have to deal with the trade-off between energy allocation towards somatic growth, mainly to reduce predation pressure, or energy storage, increasing their starvation tolerance. Plaice larvae at pre-metamorphosis, especially stage 2 individuals, appeared to favour growth, displaying very low amounts of TAG reserves and a high growth rate, although this was highly variable between individuals. This heterogeneity in growth rate could come from the different condition levels observed in stage 2 larvae. Indeed, stage 2 larvae had the highest proportion of individuals in poor condition as depicted by their high proportion of degraded and intermediate condition grades. This observation could illustrate the critical period hypothesis stated by Hjort [3], where first feeding larvae without energy reserves are more impacted by starvation and experience peak mortality. Later, stage 3 larvae which have passed through the critical period will start to accumulate energy, partly in the form of liver vacuoles. It is during this development stage that the growth rate was the highest with fewer variations due to a lower proportion of unhealthy individuals. Larvae with enough reserves will then start their metamorphosis, reducing their growth rate compared to pre-metamorphosis stages. The high TAG proportion for stage 4 larvae allows us to rule out the possibility of a decline in growth rate observed during the same period due to a poorer condition since energy storage occurs in good health [62]. This was confirmed by histology which showed a majority of healthy individuals, with numerous and wide liver vacuoles. A switch in the energy allocation strategy was thereby highlighted when larvae enter metamorphosis (at a length of 9 mm in our case).

Since the quantity of lipid reserves is particularly important for plaice larvae to withstand starvation during metamorphosis, this shift could be considered as a second critical period (after the one of exogenous feeding) for larval survival and recruitment success. Monitoring larval abundances over several years, focusing on critical periods of development, could help to develop a larval recruitment index and better understand fluctuations in adult populations. Moreover, larval abundances and condition indices should be coupled for critical periods. This approach would make it possible to estimate the number of individuals with the best chances of settlement as juveniles thanks to the percentage of individuals in good condition, and would allow to detect effects of environmental variations over years on survival probabilities.
